# Catheter-related urinary nosocomial infections in intensive care units: An epidemiologic study in North of Iran 

**DOI:** 10.22088/cjim.8.2.76

**Published:** 2017

**Authors:** Mohammad Sadegh Rezai, Masoumeh Bagheri-Nesami, Attieh Nikkhah

**Affiliations:** 1 Infectious Diseases Research Center with Focus on Nosocomial Infection, Mazandaran University of Medical Sciences, Sari, Iran.

**Keywords:** Epidemiology, Nosocomial infections, Urinary catheter

## Abstract

**Background:**

Urinary tract infection (UTI) is one of the most common infections in developing countries. The aim of this study was to investigate the incidence of nosocomial catheter- associated UTI and its related factors in hospitalized patients in intensive care units of hospitals affiliated to Mazandaran University of Medical Sciences, in 2014.

**Methods:**

This cross-sectional study was conducted on patients who were admitted in hospitals and urinary catheterization was performed for them. Beds of intensive care units were followed-up for the occurrence of catheter-associated UTI for 14 months. Data were analyzed using SPSS Version 16.

**Results:**

Our results showed that of the 1409 patients (11648 catheter - days), the incidence of catheter-related UTI was 18.2% (among 256 individuals) equals to 21.987 per 1,000 catheter - days. E. coli was the most important cause of UTI. The results show that the history of the underlying disease, duration of catheterization and perineal washing were significantly associated with the incidence of UTIs.

**Conclusion:**

The findings of this study show a high incidence of UTIs caused by catheters in ICU. The incidence of this infection increased hospital length of stay and hospital cost. It seems that the necessary use of urinary catheters and its reduced duration use can be effective in decreasing this incidence.

Nosocomial infections are defined as infections that occur within 48 to 72 hours after admission and a week after discharge; they do not include previously contacted or incubated infections at the admission time (1). Urinary tract infection (UTI) is the most common infection in developing countries (2). UTI is caused by blood and pathogens that enter the space surrounding the urethra through the perineum, the digestive system, or urinary catheter (3). UTI comprises more than 40% of nosocomial infections in the United States every year, and most of these infections are associated with urinary catheterization (4). In a study conducted in Iran, 30.9% of ICU patients with nosocomial infections had UTI (5). In a study conducted in Tehran, UTI affected 18.7% of patients (6). The most common bacteria causing UTI are *Escherichia coli* and *Pseudomonas aeruginosa* (7). The increasing incidence of UTI in ICUs is affected by the high rate of urinary catheterization, frequent contact with health care workers, and increased resistant pathogens (8). Increasing bacterial resistance to a variety of antibiotics leads to increased mortality and morbidity and prolonged hospital stay following the acquisition of nosocomial infections. Excessive use of antibiotics wastes financial resources, which account for 20% to 50% of the total hospital medication costs. More than half of the hospitalized patients are treated with antibiotics; yet more than 50% of all antibiotics administered are used for inappropriate durations (9). 

In recent years, certain measures have been taken to control nosocomial infections, including establishing nosocomial infection committees, frequent culture from different parts of the hospital, training medical employees, use of modern infectious waste disposal methods, and rinsing hands before surgical procedures (10). Nevertheless, nosocomial infections continue to cause numerous problems in the process of treatment, including prolonged hospital stay, increased medicine use and increased laboratory costs. The diversity of infectious agents, medical interventions, excessive use of a wide range of antibiotics, and the importance of prevention, diagnosis, and treatment are among the main reasons for studying the rate of nosocomial infections (11). 

To the best of our knowledge, no epidemiological study in this area has been conducted in Mazandaran, hence, the present study was conducted to investigate the incidence and factors related to nosocomial infections caused by urinary catheterization among the ICU patients of hospitals affiliated to Mazandaran University of Medical Sciences, the results can be compared to international statistics and problems in service-provider systems be identified to pave the way for logical solutions. 

## Methods

This descriptive- analytical study was conducted in the ICUs of hospitals in Mazandaran after obtaining permission from Mazandaran University of Medical Sciences. The study setting consisted of intensive care units (CCU, BICU, NICU, PICU, and ICU) containing a total of 256 beds. The study population included hospitalized patients who had undergone urinary catheterization. ICU beds were monitored daily for catheter-induced urinary infections for 14 months. Demographic and medical details of patients and clinical and laboratory symptoms of urinary infections and associated factors were recorded on nosocomial urinary tract infection checklists. These forms contained specific clinical and laboratory protocols for nosocomial infection diagnosis. The final diagnosis was necessarily based on these guidelines. The questionnaire has been used in various studies (12-15). Symptoms associated with UTI such as fever (increased body temperature equal to or greater than 38 °C), suprapubic tenderness or urinary urgency with positive urinary culture (CFU/mL ≥ 10^5^) (16), antibiogram reports and culture and types of antibiotics were recorded. Culture and antibiogram were performed according to microbiology (17, 18) and CLSI standards (19). Study exclusion criteria were fever and UTI symptoms 48 hours before admission (20). Sterile urinary samples were taken from midstream urine (16). Data were reviewed on a weekly basis by a project assistant and followed-up and revised (if necessary) using the patient's data as recorded. Statistical analysis was performed in SPSS Version 16 using descriptive statistics such as mean, frequency and percentage, and inferential statistics such as chi-square and t-tests. 

## Results

Of these 1409 cases (11648 catheter-day), 256 (18.2%) had catheter-induced UTI equivalent to 21.978 cases per 1000 catheter-day. The mean age of infected and non-infected patients was 64.503±18.66 years and 61.265±19.471 years, respectively. T-test showed a significant difference between age and time of catheterization UTI (P=0.015, 0.001, respectively) (table 1). 

**Table 1:** Factors affecting the catheter-related urinary tract infections in ICU in Northern Iran

**Variable**	**Urinary tract infection**		**Odds ratio**	**Confidence interval**	**P-value**
	**yes**	**No**			
Age	64.503±18.66	61.26±19.47	-	0.619-5.857	0.015
Time of catheterization	11.67 ± 17.65	7.69 ± 7.29	-	1.739-6.218	0.001
**Gender**					
Male Female	116(14.7%) 140(22.7%)	675(47.9%) 478(33.9%)	0.587	0.447–0.771	0.001
**Perineal ** **care**					
Yes No	229(18.3%) 11(0.9%)	875(69.4%) 145(11.5%)	3.450	1.837–6.477	0.001
**History of underlying disease**					
Yes No	131(10.5%) 106(805%)	476(38.2%) 534(42.4%)	11.386	1.043–1.842	0.004
**Antibiotic therapy before catheterization**					
Yes No	126(9.7%) 115(8.9%)	577(44.5%) 478(36.9%)	0.908	0.686-1.201	0.004

The results showed a significant relationship between UTI and history of underlying diseases, duration of catheterization, and perineum rinsing method. No significant relationship was found between UTI and the use of antibiotics before catheterization (table 1). According to the results, *E. coli* was the main agent for urinary infection (69 patients, 34.9%), followed by *Klebsiella* (29 patients, 15.3%), *Pseudomonas aeruginosa* (18 patients, 9.5%), and miscellaneous (82 patients, 40.3%). 

Samples of *E. coli* isolated from patients were highly resistant to third generation cephalosporin and aminoglycosides. The pattern of antibiotic resistance and susceptibility of a number of UTI-causing bacteria is shown in table 2.

**Table 2:** Antibiotic resistance patterns of bacterial strains isolated from urine in intensive care units in northern Iran

**SusceptibilityAntibiotic**	**E. coli**		**Klebsiella pneumonia**		**Pseudomonas aeruginosa**	
	**R**	**S**	**R**	**S**	**R**	**S**
Amikacin	2	8	4	2	4	2
Imipenems	18	9	5	9	7	5
Gentamicin	15	21	7	10	8	3
Ceftizoxim	17	15	4	5	4	2
Ceftriaxon	26	9	31	6	6	2
Ciprofloxacin/	19	9	-	10	9	4
Cefexim	5	7	29	1	4	2
Co-trimoxazole	25	10	7	4	7	3
Ampi/Sul	11	1	8	19	6	-
Amoxicillin	2	1	1	3	-	1

## Discussion

According to our findings, the catheterization rate was 18.2%. This ratio was 15.1% and 17% in other studies conducted in Iran (21, 22). In investigations conducted in other countries, this was 8.9 (23), 8.3 (24), and 9.6 (25) cases per 1000 catheter-day. As a consequence, compared to other studies, catheter-acquired UTI in Iran is twice as frequent as in other countries indicative of its high rate. The quality of healthcare personnel actions is highly important in the mitigation of nosocomial infections. A study conducted in Iran showed that nurses lack adequate knowledge of nosocomial infection control (26). The high incidence rate of catheter-induced UTI in Iran demands training programs focused on performance feedback. In the present study, women tend to have more UTIs than men. Similarly, studies by Kolawole (27) and Laupland (25) also showed a high incidence rate of UTI in women. The short urethra and its short distance to the rectum in women could explain this difference (28). The results obtained showed a direct relationship between age and UTI. The findings a study by Rafiei showed an increase prevalence rate of UTI with aging (29). In the current investigation, prolonged duration of catheter use led to increased rate of UTI. Other research also confirmed these results (30). Bacteria may gain entry into the urinary tract via catheter insertion site. Catheterization causes the biofilm development between catheter and urethral mucus, thus, preparing the environment for bacterial attack and proliferation (31). The risk of infection increases by 3% to 10% on a daily catheter-use (27). It is essential to thoroughly observe the aseptic technique during catheterization, consider alternatives to catheterization, and reduce the duration of catheter use (32). 

In this study, a significant correlation was found between UTI and the presence of an underlying disease. Furthermore, in this paper, diabetes was the most common underlying disease in patients. A study by Shah showed that diabetic patients experienced increased rate of infection and infection-related mortality (33). In another study showed that diabetic patients were found to enhance the risk for UTI, of which glucosuria and impaired immune system were induced (34). Nonetheless, reduced sensitivity and changes in bladder distention due to impaired autonomic nervous system in diabetic patients could also cause urinary stasis and increased risk of UTI in these patients (35). In the existing study, daily rinsing of the perineum led to a reduced UTI. Tsuchida et al.’s study showed a relationship between daily rinsing of the perineum and UTI. They also proposed fecal incontinence as a major risk factor in UTI incidence in patients with urinary catheterization (36). Regular rinsing of the urinary tract and perineal washing is essential, especially in patients with fecal incontinence to reduce UTI. 

In the ongoing study, the administration of antibiotics before catheterization had no effect on reducing UTI. In contrast, Crouzet et al. reported different results. In their study, the administration of antibiotics for treatment of infections in places other than urinary system before and during catheterization reduced catheter-induced UTI; yet the author did not consider this to support antibiotic therapy (32), since the non-essential administration of antibiotics can lead to emergence and development of antimicrobial resistance (37). Administering antibiotics is recommended after culture and microbial susceptibility results (38). In this study, majority of patients did not have the history of anomalies and infection of the urinary tract, so these cases were not reported. 

In this study, *E.coli* was the most common microbial agent. This was also observed in other studies (39, 40). In contrast, in a study by Taneja et al., *Klebsiella* was the most common microbial agent for UTI, followed by *E.coli*, *Enterobacter*, and *Pseudomonas aeruginosa* (41). In our study, *E.coli* showed a high resistance to third-generation cephalosporin and gentamicin. In line with this study, another study reported the highest susceptibility of *E.coli* of the study samples to amikacin and ciprofloxacin (42, 43). This bacterium showed the highest resistance to ceftriaxone. In another study conducted in Mazandaran, the most antibiotic resistance was related to the cephalosporin group (44). In a study on children, *E.coli* showed high resistance (100%) to a number of cephalosporins (45). These results confirm that *E.coli* is becoming increasingly resistant to cephalosporin. 

*Klebsiella* showed a high resistance to ceftriaxone and cefixime in this study. Klebsiella had the highest susceptibility to ampicillin-sulbactam, ciprofloxacin, gentamicin, and imipenem. A study by Paterson et al. on *Klebsiella* showed better outcomes in patients treated with carbapenem (46). Kang et al.’s study on *Klebsiella* also showed favorable results in patients treated with carbapenem (47). Due to the high prevalence of resistance to carbapenem, rational use of this antibiotic is recommended (48). In our study, *Pseudomonas aeruginosa* showed high resistance to aminoglycosides and third-generation cephalosporin. In a study conducted in 228 hospitals in Europe, the highest resistance to antibiotics included gentamicin, ciprofloxacin, and tobramycin (49). Findings of a study in Iran showing the highest resistance to *Pseudomonas aeruginosa *was caused by ceftriaxone and ciprofloxacin (44), which was similar to the present study results. In this study, its highest susceptibility was to imipenem. A study by Karlowskey et al. showed that nearly 80% of the *Pseudomonas aeruginosa* samples isolated from ICU patients were susceptible to imipenem (50). 

In conclusion, the high incidence of catheter-induced UTI in ICUs in this study, which is twice as much as in other countries, appears too high. This infection leads to prolonged hospitalization and aggregate hospital costs. It shows that the use of urinary catheter when needed and the reduced duration of catheter may decrease UTI. According to a study, infections caused by antibiotic-resistant pathogens generally cause higher mortality rates (51). Accordingly and because of the difference in resistance of the most common urinary infections such as *E.coli* to certain cephalosporins and their susceptibility to aminoglycosides based on this province-wide study; further studies are recommended to design clinical guidelines for catheter-induced UTIs.
